# Use of the evidence base in substance abuse treatment programs for American Indians and Alaska natives: pursuing quality in the crucible of practice and policy

**DOI:** 10.1186/1748-5908-6-63

**Published:** 2011-06-16

**Authors:** Douglas K Novins, Gregory A Aarons, Sarah G Conti, Dennis Dahlke, Raymond Daw, Alexandra Fickenscher, Candace Fleming, Craig Love, Kathleen Masis, Paul Spicer

**Affiliations:** 1 Centers for American Indian and Alaska Native Health, Mail Stop F800, 13055 East 17th Avenue, Aurora, CO 80010, USA; 2 Department of Psychiatry, University of California, San Diego, 9500 Gilman Dr. #0812, La Jolla, CA 92093, USA; 3 PO Box 2405, Pagosa Springs, CO 81147, USA; 4 Peaceful Spirit ARC, 296 Mouache Street, P.O. Box 429, Ignacio, CO 81137, USA; 5 Navajo Department of Behavioral Health Services, Window Rock, AZ 86515, USA; 6 Westat, 1600 Research Blvd, Rockville, MD 20850, USA; 7 Montana-Wyoming Tribal Leaders Council, 222 North 32nd Street, Suite 401, Billings, MT 59101, USA; 8 Center for Applied Social Research, Two Partners Place, 3100 Monitor Avenue, Suite 100, Norman, OK 73072, USA

## Abstract

**Background:**

A variety of forces are now shaping a passionate debate regarding the optimal approaches to improving the quality of substance abuse services for American Indian and Alaska Native communities. While there have been some highly successful efforts to meld the traditions of American Indian and Alaska Native tribes with that of 12-step approaches, some American Indian and Alaska Natives remain profoundly uncomfortable with the dominance of this Euro-American approach to substance abuse treatment in their communities. This longstanding tension has now been complicated by the emergence of a number of evidence-based treatments that, while holding promise for improving treatment for American Indian and Alaska Natives with substance use problems, may conflict with both American Indian and Alaska Native and 12-step healing traditions.

**Discussion:**

We convened a panel of experts from American Indian and Alaska Native communities, substance abuse treatment programs serving these communities, and researchers to discuss and analyze these controversies in preparation for a national study of American Indian and Alaska Native substance abuse services. While the panel identified programs that are using evidence-based treatments, members still voiced concerns about the cultural appropriateness of many evidence-based treatments as well as the lack of guidance on how to adapt them for use with American Indians and Alaska Natives. The panel concluded that the efforts of federal and state policymakers to promote the use of evidence-based treatments are further complicating an already-contentious debate within American Indian and Alaska Native communities on how to provide effective substance abuse services. This external pressure to utilize evidence-based treatments is particularly problematic given American Indian and Alaska Native communities' concerns about protecting their sovereign status.

**Summary:**

Broadening this conversation beyond its primary focus on the use of evidence-based treatments to other salient issues such as building the necessary research evidence (including incorporating American Indian and Alaska Native cultural values into clinical practice) and developing the human and infrastructural resources to support the use of this evidence may be far more effective for advancing efforts to improve substance abuse services for American Indian and Alaska Native communities.

## Background

### Focus of this debate

Despite concerted efforts to improve alcohol and drug abuse prevention and clinical programs as well as decades of research, available information suggests that the prevalence of problematic substance use has not appreciably changed in many American Indian and Alaska Native (AI/AN) communities [1-4]. While the specific contexts, patterns, and severity of these difficulties do vary across AI/AN communities [5-9], the overall rates of problematic substance use [1,2,4,5,7,8] and related morbidity and mortality [10-12] are comparable to - or far exceed - the rates of non-AI/ANs.

Given this, an effective substance abuse treatment system is critical to address these needs in AI/AN communities. Unfortunately, while dedicated clinicians, clinical programs, tribes, and the Indian Health Service (IHS) have developed some highly innovative treatment approaches in many AI/AN communities [13-18], such services remain severely underfunded and many AI/AN communities still have limited access to substance abuse treatment services [19,20]. Indeed, epidemiological studies confirm that only a small percentage of those participants with substance use disorders received substance abuse treatment [21].

A variety of forces, both internal and external to AI/AN communities, are now shaping a passionate debate regarding the optimal approaches to improving the quality of this substance abuse service system. Changes in federal policy dating to the Nixon administration have provided AI/AN communities much greater autonomy in developing and implementing a variety of services - including substance abuse services - independent of federal oversight [22]. This is coupled with a groundswell of interest in applying the healing traditions that are integral to AI/AN cultures to address substance use and related problems [14,23-27]. Nevertheless, many substance abuse programs serving AI/AN communities continue to draw upon 12-step approaches that were originally introduced when funds for such programs were first made available in the United States in the 1960s and 1970s [27-30]. While there have been some highly successful efforts to meld the traditions of AI/AN tribes with that of 12-step approaches [31,32], some AI/ANs remain profoundly uncomfortable with the dominance of this Euro-American approach to substance abuse services in their communities [27,33]. This long-standing tension has now been compounded by the emergence of a number of evidence-based treatments (EBTs; described further below) [34] that, while holding substantial promise for improving services for AI/ANs with substance use problems (as they do for non-AI/ANs), may conflict with both AI/AN and 12-step healing traditions and may be seen as yet another imposition of alien approaches in AI/AN programs. Reinforcing these concerns is the fact that AI/ANs have rarely participated in the clinical trials to establish the efficacy of these EBTs, in part because of their reluctance to do so because of a history of substantial research abuses [33,35] as well as serious questions regarding the value of research for improving circumstances for AI/AN people [36,37]. This lack of evidence and longstanding wariness of research further contributes to the hesitancy of programs serving AI/AN communities to implement these treatments [33,38]. Efforts of policymakers to encourage (and sometimes require) the use of these EBTs in programs receiving federal and state funding [39,40] are further intensifying this debate.

Our research team is interested in studying how EBTs are perceived and used (or not used) by substance abuse programs serving AI/AN communities. However, as we prepared to start this investigation, it became clear from both our conversations with key stakeholders and our review of recent publications that this debate has become so contentious that we needed to address these issues squarely in the design of our research. We therefore convened an advisory board for a three-day meeting in September 2008 to discuss these controversies, how these controversies might threaten our data collection efforts, and methods to reduce the risks of these threats to our project. Along with our original team of University-based researchers (with expertise in clinical, cultural, and epidemiological sciences), our Advisory Board consists of experts from substance abuse programs serving AI/AN communities working in clinical, administrative, evaluative, and policymaking capacities. These experts were invited to participate based on their reputations for having pursued the development of high quality substance abuse services for AI/AN communities at the local, regional, and national levels while at least partially representing the geographic and cultural diversity of AI/AN communities. A subset of the Advisory Board then pursued the completion of this manuscript using an iterative process in which we referred to detailed meeting notes, reviewed additional literature for incorporation in the manuscript beyond that identified during the meeting, and engaged in an ongoing 'discussion' consisting of email exchanges as well as use of Microsoft Word's comments and track changes functions on serial drafts of the manuscript until we felt we had fully captured the original discussion and its implications. This subset of the Advisory Board are the authors of the manuscript. All Advisory Board members were given the opportunity to review and comment upon final drafts of the manuscript prior to its submission (and resubmissions). This paper is a summary of these discussions and addresses the following areas: additional background regarding the specific community, policy, and practice contexts for this debate; a description of the key lines of tension around approaches to substance abuse services in AI/AN communities; and a discussion regarding specific concerns about the use of EBTs in programs serving AI/AN communities and their likely influence on the dissemination process.

### Community, policy, and practice contexts

#### Community contexts

AI/ANs are a diverse and heterogeneous population. There are over 560 federally recognized tribes in a population that numbered nearly 2.5 million in 2000; over 4 million if people listing AI/AN in conjunction with other races are included [41]. The majority of the AI/AN population resides in the western United States, and is, on average, younger, less educated, and poorer than the U.S. general population [41-43]. While a greater percentage of the AI/AN population resides in rural areas than the U.S. general population (34% versus 21%), the majority of AI/ANs now reside in urban/suburban areas [44].

Also notable - after centuries of repression - is the resurgence in community interests in tribal languages and traditions, including traditional healing [26,33], though engagement in and identification with AI/AN and the majority culture vary considerably from individual to individual and community to community [45]. Recent research suggests that many AI/ANs rely on traditional healing to address alcohol, drug, and mental health problems, both independently and in combination with treatments emerging from Euro-American traditions [25]^a^. Indeed, some authors have advocated a greater reliance on traditional healing to address these mental health and substance use problems and express considerable skepticism about the utility of Euro-American-based treatments and the research methods used to develop them noting that some AI/AN academicians, service providers, and community members feel they represent another form of colonialism that is harming rather than helping AI/AN people and communities [46].

#### Policy contexts

In order to understand the substance abuse service system in AI/AN communities, it is helpful to start with its unique funding and service delivery mechanisms, which have undergone radical changes in recent years. Since 1955, the IHS has developed a health care system for AI/AN communities at no cost to those eligible. Hospitals and clinics are operated either by the IHS or by tribes.

Recent changes to this system are largely the result of the Indian Self-Determination and Educational Assistance Act (Public Law 93-638), which has given participating AI/AN tribes greater flexibility and autonomy to restructure human services. Indeed, substance abuse services have long been at the cutting edge of the trend towards greater tribal control of human services with the majority of programs operated by tribes and non-governmental tribal entities (such as urban Indian health boards) [22,47] rather than the IHS. Unfortunately, these policy changes have occurred concurrently with substantial declines in what was already inadequate funding. Funds for health care in AI/AN tribes, adjusted for medical inflation and population growth, saw steady declines throughout the 1990s [48]. This trend has continued over the last decade [47,49]. Even a record increase for IHS in 2001 was barely sufficient to keep up with medical inflation in that year [50-52]. The National Indian Health Board reported that per capita benefits for those AI/ANs receiving IHS-supported services is one-half of that for Medicaid beneficiaries and one-third of that for Veterans Affairs beneficiaries [53]. The US Civil Rights Commission has echoed these concerns [51,52]. Also troubling is the fact that funding for health services for AI/ANs has not kept pace with demographic trends. Urban Indian health boards, which were chartered by the IHS, receive very limited funding (about 1% of the IHS budget) even though the majority of AI/AN people now live in urban and suburban areas [44,54] (unpublished data, National Center for Urban Indian Health).

Understandably, programs serving AI/AN communities have responded to these real declines in funding by seeking out other sources of programmatic support. For example, the Substance Abuse and Mental Health Services Administration's (SAMHSA) Center for Substance Abuse Treatment (CSAT) has awarded more grants in recent years to tribes, tribal consortiums, and urban Indian health boards than has been typical in the past [55]. Some tribes have aggressively pursued Medicaid and third party reimbursement for health services (including behavioral health) [50,56]. In Arizona, three tribes (Gila River, Navajo, and Pascua Yaqui) function as Tribal Regional Behavioral Health Authorities, thus serving as their own Medicaid-funded behavioral health programs [57]. All of these funders are also moving towards requiring EBTs for grant funding and reimbursement for clinical services. For example, CSAT now requires that grant applicants specify the EBTs they will use for services supported through these funds, and the Oregon Health Plan is phasing in an EBT requirement for all health services, including substance abuse services [39,40,58]. Given these emerging requirements for EBT use, we expect that many AI/AN programs are developing ways to respond to these requirements, although the nature of these responses are largely unknown.

#### Practice contexts^b^

The substance abuse service system for AI/AN communities emerged out of the same federal efforts of the 1960s and 1970s that shaped their counterparts in the rest of the United States [30]. Although the development of the service system for AI/AN communities was managed somewhat separately from that for the rest of the United States - substance abuse programs were transferred from the National Institute of Alcohol Abuse and Alcoholism to the IHS in 1978 [59], and the training of counselors for these programs was (and is) often done through special training programs (*e.g.*, the Southwest Certification Board) [60], the programs that emerged similarly relied on a cadre of counselors who were trained to utilize treatment models that grew out of the 12-step movement [30].

Some believe the legacy of 12-step-trained counselors and 12-step-based treatments has impeded the acceptance and use of EBTs in substance abuse programs across the United States [61]. What is particularly notable, and distinct, about the development of the substance abuse service system in AI/AN communities was the considerable resistance to 12-step approaches to treatment, particularly from those AI/ANs most strongly connected to their Native cultures [62]. These community members felt 12-step approaches conflicted with their traditional beliefs, and it took a concerted effort by AI/ANs such as Earl L., Gene Thin Elk, and Don Coyhis to adapt these approaches for AI/ANs and to advocate for their use [31]. While some AI/ANs remain uncomfortable with 12-step approaches, these efforts have resulted in large-scale acceptance of 12-step treatments in AI/AN communities, though this is in the context of a strong emphasis on combining 12-step and traditional AI/AN practices [31].

The IHS website lists 480 behavioral health programs serving AI/AN communities [63], but there are no reliable surveillance data regarding the nature and scope of substance abuse services for AI/ANs and questions about the quality of behavioral health services for AI/ANs remain [22,64]. Novins *et al*.'s detailed study of AI adolescents admitted to a residential substance abuse program provides the most rigorously collected data [29,65]. Results show that while this particular program uses a 12-step framework for its services, it also includes a traditional healing component, utilizes cognitive behavioral therapy, and offers pharmacotherapy for comorbid non-substance use psychiatric disorders. It is unclear, however, if such blending of treatment approaches is common in substance abuse programs serving AI/AN communities.

### Evidence-based treatments for substance abuse problems

EBTs for substance abuse treatment can be divided into two broad categories, psychosocial and pharmacologic. Psychosocial treatments are largely based on behavioral and cognitive-behavioral theoretical models. Behaviorally-based treatments such as Contingency Management [66] rely on principles of operant conditioning to provide positive reinforcement (*i.e.*, rewards) for progress in treatment (most typically abstinence from substance use). Psychosocial treatments are typically provided by a psychotherapist in individual, couples, family, and group settings. The number of sessions vary considerably across EBTs, with some involving as few as two sessions (*e.g.*, Motivational Interviewing [67]) while others involve as many as 15-20 sessions (*e.g.*, Behavioral Couples Therapy [68] and Relapse Prevention Therapy [69]. Pharmacologic treatments for substance use problems involve the use of medications for the treatment of withdrawal syndromes in the initial stage of stopping the chronic use of an addictive substance (including alcohol and opiods), [70] medication to reduce the risk of relapse [71], and medications to treat comorbid psychiatric conditions that may contribute to substance related problems (including mood disorders such as Major Depression) [72,73]. See Table 1 for a list of selected EBTs for substance use problems.

**Table 1:** Selected examples of evidence-based treatments for substance use problems

Evidence-based treatment	Brief description and citation
**Psychosocial Treatments - Behavioral**	

• Contingency management	Provide positive reinforcement (*i.e.*, rewards) for progress in treatment (most typically abstinence from substance use) [66].

**Psychosocial Treatments - Cognitive Behavioral**	

• Motivational interviewing/ motivational enhancement therapy	Focuses on facilitating behavioral change by helping individuals to explore and resolve ambivalence towards treatment and become committed to addressing their substance use problems [67].

• Behavioral couples therapy	Focuses on building an abstinence-supporting relationship between the person who is abusing substances and his or her partner [68].

• Relapse prevention therapy	Teaches individuals with substance addiction a number of specific skills to reduce the risk of relapse [69].

**Pharmacologic Treatments**	

• Medication for relapse prevention	The use of medications to help prevent relapse of substance use problems, such as naltrexone, methadone, and buprenorphine [71].

### Lines of tension around substance abuse services for AI/AN communities

**Figure 1:**
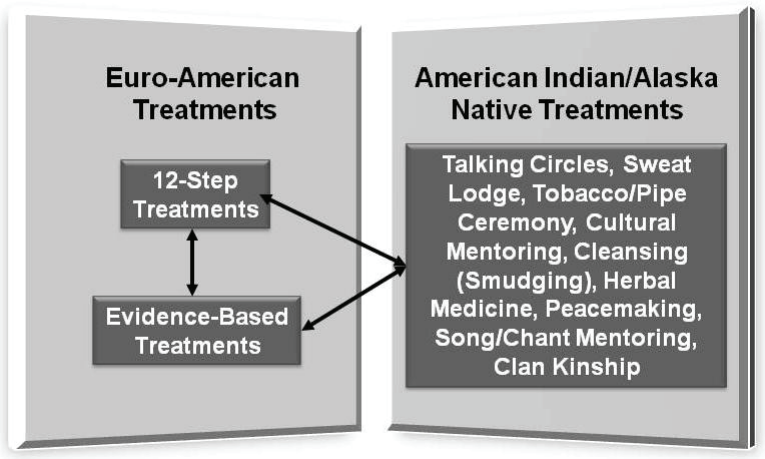
**Lines of tension in substance abuse services for American Indian/Alaska Natives**. This list of treatments, generated by our Advisory Board, provides a partial listing of traditional American Indian/Alaska Native treatments. These practices are typically named in the tribal languages; specific procedures also vary across tribes.

Figure 1 represents the key lines of tension our Advisory Board identified around the use of EBTs in substance abuse programs serving AI/AN communities. The two Euro-American sets of practices, 12 step and EBTs, now form the basis of most substance abuse services offered in the United States, and the tensions between them have been described previously [61]. For example, the landmark 1998 Institute of Medicine (IOM) report 'Bridging the Gap Between Practice and Research: Forging Partnerships with Community-Based Drug and Alcohol Treatment' [28] identified a number of factors that the authors felt impeded the transfer of knowledge between researchers and clinical programs. These factors included those related to research (*e.g.*, the study of interventions that were impractical in real-life settings), clinical practice (*e.g.*, negative attitudes towards research), and policy (*e.g.*, policies that bar the use of specific EBTs) [28]. Indeed, many of CSAT's, National Institute of Alcohol Abuse and Alcoholism's (NIAAA), and National Institute of Drug Abuse's (NIDA) efforts to promote the use of EBTs, including CSAT's Addiction Technology Transfer Centers [74,75] and NIDA's 'Clinical Trials Network,' [76,77] were a direct response to the findings and recommendations of this and other reports.

Given the historical roots of substance abuse programs in AI/AN communities described above, these tensions and the national efforts to address them are clearly relevant to understanding programmatic attitudes towards and use of EBTs. However, it is also important to account for the considerable tensions between the two Euro-American practices and practices that emerge from AI/AN traditions. Several decades of experience have allowed the AI/AN substance abuse treatment community to address the tensions between 12-step practices and AI/AN practices (and have resulted in several innovative approaches noted previously). However, efforts to promote the use of EBT's are relatively new, and the range of responses by substance abuse programs serving AI/AN communities is largely unknown. Given our collective experiences in working with a number of these programs, we expect that there is a considerable range of responses, including wholesale adoption of EBTs with minimal adjustments to account for 12-step and AI/AN traditional practices, selective adoption of specific elements of EBTs which are melded with 12-step and/or AI/AN practices, and ongoing resistance to the use of EBTs in any form.

Further complicating the incorporation of EBTs into these substance abuse programs are basic questions that some AI/AN academics and service providers have regarding the validity and ultimate value of the scientific process when applied to the needs of their communities, seeing this as another imposition of non-AI/AN worldviews on their communities [26,36,78,79]. Indeed, tribes, tribal organizations, and organizations representing other diverse communities have successfully pressed for changes in the language regarding EBTs in CSAT grant announcements [80] as well as an alternative, non-EBT pathway for approval of AI/AN treatments for Medicaid reimbursement in Oregon [81]. These efforts can be linked to a larger, 'Practice-Based Evidence Movement,' [82] which emphasizes the value of systematic evaluation of interventions within community practice settings and is usually described as a complement of [82] - and sometimes as an alternative to [26,79] - EBTs. In AI/AN communities, the Practice-Based Evidence movement further emphasizes that AI/AN treatments should have primacy over EBTs, and that such Euro-American treatments should be integrated into AI/AN treatments rather than the reverse [26,79].

However, others have suggested that there are important parallels between the scientific process and traditional AI/AN ways of understanding themselves and the world around them (something that has been proposed by several scholars for indigenous people more generally [83]); others have suggested that there is value in harnessing the scientific process for the benefit of AI/AN people [84]. Indicative of the complexity of community sentiments regarding science are the facts that members of our Board are personally working with several communities to develop manualized interventions for testing, and at least two AI/AN programs participate in NIDA's Clinical Trials Network [85]. However, even in these situations, the barriers for the effective use of EBTs are likely substantial. We will now consider those we expect are likely to be particularly salient.

### Discussion of specific concerns regarding the use of EBTs in programs serving AI/AN communities

Based on our review of the available literature and our experiences in the field, we identified a number of issues that we felt were likely impacting attitudes towards, and use of, EBTs in substance abuse programs serving AI/AN communities. We then classified these issues based on Greenhalgh *et al*.'s [86] summary of the factors associated with the successful dissemination of innovations into clinical practice. These factors were subsequently highlighted in the Institute of Medicine's report 'Improving the Quality of Health Care for Mental and Substance-Use Conditions' [87] and provide a useful rubric for classifying the issues we identified and for discussing the impacts we hypothesize they are having on the use of EBTs in these substance abuse programs. The most relevant factors are discussed below and summarized in Table 2.

**Table 2:** Factors associated with dissemination of innovations and how these factors likely influence use of EBTs^a^

Factor	**Likely Direction of Influence**^**b**^
Characteristics of the innovation. Innovation more likely to be adopted if it:	
• Is compatible with adopters' values, norms, needs.	↓↓↓
• Is simple to implement.	↓↓
• Can be adapted, refined, modified for adopters' needs.	↓↓↓
• Is accompanied by easily available or provided knowledge required for its use.	↓↓↓

Sources of communication and influence. Uptake of innovation influenced by:	
• Structure and quality of social and communication networks.	↓
• Similarity of sources of information to targeted adopters; *e.g.*, in terms of socioeconomic, educational, professional, and cultural backgrounds.	↓

External influences. Uptake of innovation influenced by:	
• Policy mandates.	↑↓ (attitudes), ↑↑↑ (use)

Linkages among the components. Innovation more likely to be adopted if there are:	
• Formal linkages between developers and users early in development.	↓↓↓
• Effective relationships between any designated "change agents" and targeted adopters.	↓

Characteristics of individual adopters	
• General and context-specific psychological traits.	↑↓
• Finding the intervention personally relevant.	↑↓

Structural and cultural characteristics of potential organizational adopters. Innovation more likely to be adopted if organization:	
• Has effective data systems.	↓↓
• Is "ready" for change because of ...available time and resources for change, and capacity to evaluate innovation's implementation.	↓↓↓

The uptake process. Innovation more likely to be adopted with:	
• Funding.	↓↓↓ (Tribal/IHS), ↑↑↑ (EBT-specific)
• Adaptation and reinvention.	↓↓↓
Programmatic Priorities. Innovation more likely to be adopted if it:^c^	
• Is consistent with the programmatic priorities of the adopter.^c^	↓↓

**Notes**. ^a^list adapted from that developed by Greenhalgh *et al*., 2004 [86].

^b^likely direction of influence refers to our perceptions of how these particular factors are affecting the dissemination process in substance abuse treatment programs serving AI/AN communities as follows: ↑↑↑ - strongly supportive of the dissemination process; ↑↓ - mixed/neutral; ↓ - somewhat negative; ↓↓ - negative; ↓↓↓ - strongly negative.

^c^an additional factor identified by our Advisory Board but not included in Greenhalgh *et al*.'s summary.

### Characteristics of the innovation

Our group concluded that the characteristics of many of the EBTs themselves and their potential lack of fit with the values of providers and the communities they serve [88] are likely a major factor in limiting their dissemination to substance abuse programs serving AI/AN communities. In particular, we believe there is a strong perception that many EBTs are not in and of themselves culturally appropriate for use with AI/ANs. The lack of a spiritual component to the vast majority of EBTs - a core component of 12-step approaches that has likely contributed to their successful adaptation for use in AI/AN communities - may also reduce the likelihood of their use. An additional concern is that many EBTs are too rigid to support implementation in substance abuse programs with limited human, infrastructural, and financial resources.

Cultural adaptation of EBTs to better match consumer and community preferences and programmatic adaptation to better match programmatic capabilities were issues the Advisory Board often returned to in our discussion. While such adaptations seem a particularly compelling approach to increasing the likelihood of the use of EBTs, the success of such approaches in the available literature for behavioral health interventions is mixed at best, with some successes reported [89,90], but also some failures [91,92]. Indeed our Board noted two contrasting examples in this regard. Multisystemic Therapy and Strategies, in which research identified the importance of maintaining intervention fidelity to assure its effectiveness [93] led its developers to design a dissemination model that provides intensive training and ongoing supervision of clinicians [94]. In contrast, Motivational Interviewing has been subject to some research and several program development efforts to develop culturally-adapted manuals and treatment guidelines for use with AI/ANs [17,95]. However, Motivational Interviewing was perceived as a rare exception in this regard. Indeed, our Board is encouraged by the growing body of literature focused on identifying 'core elements' of EBTs that are important to retain to maintain their effectiveness [96-98]. Such research offers great promise for guiding thoughtful adaptation efforts and our Board recommended that these efforts should be extended to include AI/AN communities.

In the end our Board hypothesized that the lack of dissemination of clear, written guidance, or easily understood process models about how to effectively adapt most EBTs for these programs and communities decreases the likelihood of their use. Indeed, while we believe emerging models for cultural adaption of interventions may be useful for programs serving AI/AN communities [89,99], we concluded that these core characteristics of EBTs themselves present major challenges for their use in programs serving AI/AN communities.

### Sources of communication and influence

While our group did not identify this as a major issue for substance abuse programs serving AI/AN communities, it is certainly true that most of these programs have historically operated in an environment geographically, organizationally, and socially isolated from clinical programs serving other communities in the United States. Clinical programs serving AI/AN communities have had unique funding sources, training programs that have focused on the needs of AI/AN providers, certification boards, and their own networks of meetings and publications. The One Sky Center was funded by SAMHSA to function as an Addiction Technology Transfer Center specifically for programs serving AI/AN communities (though this funding was ultimately discontinued, the Center continues to operate as part of the Oregon Health and Sciences University [100]). While we believe this separation has eroded in recent years, it has not entirely disappeared and likely reduces the opportunity of programs to learn about and consider new, emerging EBTs.

### External influences

As we discussed previously, the mandates for use of EBTs by a variety of funders has created considerable controversy within the AI/AN substance abuse treatment community. While this has contributed to a highly charged environment that creates significant challenges for research in this area, more and more substance abuse programs are seeking funding that brings with it requirements to use EBTs. Funding is generally seen as one of the most important but least changeable factors impacting EBT implementation [101]. Therefore, we expect these mandates are increasing the use of EBTs in these programs while creating both positive and negative attitudes towards their use.

### Linkages among the components

Here Greenhalgh *et al. *[86] are referring to the connections between developers and users of the innovation. As was the case for sources of communication and influence, we expect that the historical separation of AI/AN clinical programs makes such linkages less likely, and this results in a negative influence on both attitudes towards and use of EBTs. As we noted previously, we do believe that a handful of programs are participating in intervention research projects, but we suspect that these are the exception rather than the rule.

### Characteristics of individual adopters

As with substance abuse programs in general, the characteristics of organizational leaders and front-line clinicians are likely quite variable in programs serving AI/AN communities. For example, in some programs we are familiar with, the organizational leaders are substance abuse professionals. In other programs, however, these leadership positions may be filled by individuals with strong political connections but limited expertise in substance abuse services. Similarly, the educational levels, training, and experience with manualized treatments among front-line clinical staff is likely variable as well. Given the evidence that local leadership at the clinic or team level, along with providers' education level, experience, and level of professional development can impact provider attitudes toward adopting EBTs [102,103], these variations no doubt influence the use of EBTs in individual programs. Indeed, our Board expects that workforce issues are one of the major barriers to the use of EBTs in these programs. However, because we believe the variability in individual adopter characteristics across programs is likely substantial, their overall impact for programs serving AI/AN communities is difficult to predict.

### Structural and cultural characteristics of potential organizational adopters

Critical here are programmatic resources - human, infrastructural (including information technology), and fiscal - that are necessary to learn about EBTs and receive the necessary training for their use as well as the tools to implement them and evaluate their impact. Given that many programs serving AI/AN programs are severely constrained in these areas, we expect that these factors have a strong negative influence on both attitudes towards and use of EBTs.

### The uptake process

The current uptake process likely has a mixed impact on attitudes towards and use of EBTs in substance abuse programs serving AI/AN communities. As noted above, the real declines in IHS funding likely have a strong negative impact on the use of EBTs as the resulting human and infrastructural limitations make it difficult to implement and evaluate EBTs. In contrast, the availability of EBT-focused funding likely has a strong positive impact, at least among the programs that successfully compete and/or qualify for these funds. Even with increased funding, the perceived inflexibility of these EBTs and lack of guidance regarding their adaptation likely have a strong negative influence on the dissemination process.

### Programmatic priorities

Finally, our group identified an additional factor that did not easily fit into the Greenhalgh *et al. *[86] framework - that the focus on EBTs is likely perceived as misplaced by many individuals and programs working in this area as it potentially neglects some of the basic foundations for quality substance abuse clinical services. These foundations include effective workforce development and stability, well-designed (and maintained) facilities, a modern information technology infrastructure, and improved access to services for community members in need. There are very few programs that have active consumer- or community advocate-involvement. Thus, we hypothesize that EBTs are of lower priority for these substance abuse programs given these other challenges they face in maintaining the services they currently provide. This likely has a strong negative influence on the dissemination process.

In summary, we believe that the vast majority of these factors - particularly those that are internal to these substance abuse programs - are likely limiting the dissemination of EBTs to substance abuse programs serving AI/AN communities. And it is primarily external factors - those of policy and funding - that are likely facilitating the dissemination process.

Our analysis provides a preliminary explanation for the strong sense of concern and controversy we encountered as we began our investigation. Indeed, given the sovereign status of tribes and their power to make decisions for their communities, a substantial reliance on external factors for promoting dissemination is, at best, seriously flawed as a strategy for effective dissemination.

### Summary

The controversy around EBTs and substance abuse services for AI/ANs is concerning for many reasons. Perhaps of most importance is that this controversy is creating divisions among key stakeholders that should be more strongly aligned if we are to improve the quality of services for AI/ANs with substance use problems. The initiatives to increase the use of EBTs in substance abuse programs have certainly grown out of a strong, nationwide and multidisciplinary desire to improve the quality of services provided to Americans with substance use problems. While the goals of these efforts are certainly laudatory, the unique community, policy, and practice contexts that appear to complicate these efforts in AI/AN communities have yet to be adequately explored. Indeed, in our conversations we were all struck by the fact that the perceived focus of these efforts is to promote EBTs rather than Evidence-Based Practice - *the integration of best research evidence with clinical expertise and patient values *[104]. An explicit shift to promoting evidence-based practice rather than EBTs might allow for a broader and more constructive conversation around improving the quality of substance abuse services in AI/AN communities. Indeed, broadening the conversation to include building the necessary research and practice evidence, developing the human, infrastructural, and fiscal resources to support the use of this evidence, and more careful thought about incorporating patient (and AI/AN cultural) values into clinical practice, may be far more attractive - and much less controversial - than the current approach. For example, careful consideration of the implications of the unique patterns of substance use in specific AI/AN communities, such as the high rates of abstinence from alcohol use [7,8], may result in a more acceptable process for selecting and adapting existing EBTs - as well as developing AI/AN-specific interventions. Our analysis of the specific concerns about the use of EBTs in substance abuse programs strongly suggests that it is largely factors external to these programs that are driving the move towards the use of EBTs. Unless there are changes in internal factors, dissemination efforts will continue to falter.

It may also be helpful to return to the IOM's original recommendations to improve connections between researchers and service organizations [28]. These 12 policy recommendations spanned the following six key areas: 1) linking research and practice; 2) linking research findings, policy development, and treatment implementation; 3) knowledge development; 4) dissemination and knowledge transfer; 5) consumer participation; and 6) community-based research collaboration. The first, fifth and sixth areas - linking research and practice as well as consumer participation and community-based research collaboration - seem particularly important areas if we are to shift from an externally-mandated to an internally-driven process of change. Such foci are also consistent with Greenhalgh *et al*.'s [86] emphasis of the importance of linkage of the 'knowledge purveyors' (*e.g.*, researchers), the 'change agency' (*e.g.*, funders of services), and the 'user system', not only at the intervention dissemination and implementation stages, but at the intervention design stage as well. Aarons *et al*.'s [101] findings regarding the public mental health system in San Diego further underscore the importance of integrating multiple stakeholders' perspectives, input, and governance in order to better understand and address the need to implement more effective services.

And while the historical, political, cultural, and infrastructural contexts for substance abuse services for AI/AN communities are unique, many of the concerns we identified (and potential solutions) are shared by non AI/AN communities. These include concerns about the lack of flexibility of many EBTs for use with clients who are diverse both clinically and culturally as well as in resource-poor clinical programs (characteristics of the innovation), the heavy reliance on external mandates for driving program change (external influences), and limited programmatic resources (structural and cultural characteristics of potential organizational adopters). While these shared concerns certainly raise the possibility of enhancing national approaches to improving substance abuse services so that they are more effective for programs serving both AI/AN and non-AI/AN communities, it is important that we not lose sight of the distinctive characteristics of AI/AN communities and the programs that serve them and that these will likely require the development of tribally-specific approaches (*e.g.*, the resurgence in interests in AI/AN traditions and knowledge as well as the importance of tribal sovereignty). Similarly, the issues noted here for substance abuse services in AI/AN communities are likely comparable for mental health services as well as services for chronic health conditions that include cognitive behavioral techniques for supporting behavior change (*e.g.*, the Healthy Heart Program for reducing the risks of diabetes-related cardiovascular disease [105]). An exploration of these issues for these other aspects of the health care systems serving AI/AN people would be a worthwhile exercise.

The extent to which these issues are comparable to other indigenous communities (*e.g.*, the Maori people of New Zealand, First Nations people in Canada, Khosian people of Southern Africa) is more complex and intriguing than it might appear at first glance. As these groups share parallel histories of European colonization and control, we would certainly expect some similarities in the perceptions and use of EBTs. For example, publications regarding substance abuse and mental health treatment for the Maori suggest there are indeed similar concerns about the cultural adaptation of standard treatments [106,107] and there are efforts such as the Healing our Spirit Worldwide that aims at linking the efforts of indigenous groups internationally [108]. However, it is equally important to note each of these indigenous communities is also quite distinct with important differences in their histories and contemporary circumstances. For example, tribal sovereignty is an important factor in how these issues have unfolded for AI/AN people as we have noted in this paper, but the legal status of indigenous peoples varies enormously from country to country [109-111]. Finally, the countries within which these indigenous communities are embedded have markedly different health care systems and have varying approaches to substance abuse treatment [112,113]. It is likely that the complex interactions of history, contemporary status, and health care systems result in important differences in how EBTs are perceived and used. Therefore the issues described in this debate should be extended to other indigenous populations with considerable caution.

This controversy also complicates research efforts in this area - including our own. However, we came out of our Advisory Board discussions with a much stronger understanding of this controversy and how best to proceed to assure that our research is an accurate reflection of the environment for dissemination of EBTs to substance abuse programs for AI/AN communities. This movement from expert opinion (as reflected in this paper) to empirical evidence promises to illuminate, enhance, and provide a more solid foundation in efforts to improve the quality of substance abuse services for AI/AN communities, and enrich our national conversations regarding EBTs and Evidence-Based Practices for all Americans.

### Endnotes

^a^Euro-American treatments include those from the biomedical and behavioral sciences as well as those emerging from other aspects of Euro-American culture (*e.g.*, 12-step programs).

^b^There is no scholarly history of the substance abuse services in AI/AN communities. The information here was garnered from the contributions of Gordon Belcourt, Raymond Daw, Candace Fleming, and Kathy Masis to this manuscript, all of whom were active participants in the development of these services.

## Competing interests

Gregory A. Aarons is an Associate Editor of Implementation Science. All decisions on this manuscript were made by another senior editor. The authors declare that they have no other competing interests.

## Authors' contributions

DN is responsible for the conception and design of the study. He chaired the advisory board discussions that identified the central issues discussed in this paper and was responsible for compiling these discussions and developing an overall framework for their presentation. DN, GA, SC, DD, RD, AF, CF, CG, KM, and PS are members of the advisory board for this project and were involved in the initial discussions that identified the central issues discussed in this paper. They were involved in drafting and revising this manuscript and have given final approval of the version submitted for review.
